# Validation of the Spanish version of the Electronic Facial Palsy Assessment (eFACE)

**DOI:** 10.1007/s00405-023-08132-4

**Published:** 2023-08-03

**Authors:** Teresa Mato‑Patino, Isabel Sánchez‑Cuadrado, Julio Peñarrocha, José Manuel Morales‑Puebla, Jesús Díez‑Sebastián, Javier Gavilán, Luis Lassaletta

**Affiliations:** 1 Department of Otolaryngology, La Paz University Hospital, Madrid, Spain; 2 Department of Preventive Medicine, La Paz University Hospital, Madrid, Spain; 3 IdiPAZ Research Institute, Madrid, Spain; 4 Biomedical Research Networking Centre On Rare Diseases (CIBERER), Institute of Health Carlos III (CIBERER-U761), Madrid, Spain; 5 PhD Program in Medicine and Surgery, Autonomous University of Madrid, Madrid, Spain

**Keywords:** eFACE, Validation, Facial palsy, Facial nerve, Spanish, Clinical tool

## Abstract

**Purpose:**

The clinician-graded electronic facial paralysis assessment (eFACE) is a relatively new digital tool for assessing facial palsy. The present study aimed to determine the validity and reliability of the Spanish version of the eFACE.

**Methods:**

Forward–backward translation from the original English version was performed. Videos and photographs from 65 adult patients with unilateral facial paralysis (any severity, time course, and etiology) were evaluated twice by five otolaryngologists with varying levels of experience in facial palsy evaluation. Internal consistency was measured using Cronbach’s α and the intra- and inter-rater reliability were measured using intraclass correlation coefficient. Concurrent validity was established by calculating Spearman’s rho correlation (ρ) between the eFACE and the House–Brackmann scale (H–B) and Pearson’s correlation (r) between the eFACE and the Sunnybrook Facial Grading System (SFGS).

**Results:**

The Spanish version of the eFACE showed good internal consistency (Cronbach’s α > 0.8). The intra-rater reliability was nearly perfect for the total score (intraclass correlation coefficient: 0.95–0.99), static score (0.92–0.96), and dynamic score (0.96–0.99) and important-to-excellent for synkinesis score (0.79–0.96). The inter-rater reliability was excellent for the total score (0.85–0.93), static score (0.80–0.90), and dynamic score (0.90–0.95) and moderate-to-important for the synkinesis score (0.55–0.78). The eFACE had a very strong correlation with the H–B (ρ =  – 0.88 and  – 0.85 for each evaluation, *p* < 0.001) and the SFGS (*r* = 0.92 and 0.91 each evaluation, *p* < 0.001).

**Conclusion:**

The Spanish version of the eFACE is a reliable and valid instrument for assessment of facial function in the diagnosis and treatment of patients with facial paralysis.

## Introduction

Facial palsy (FP) is a common disease caused by the damage of the seventh cranial nerve. FP can lead to impaired facial movement, disfigurement, and other functional limitations, such as eye complications, eating problems, and difficulty socializing [1–3].

A standardized facial function assessment is essential for the management of FP. Nowadays, there is not a generally accepted objective system to evaluate FP. Although facial grading scales are subjective and user-dependent instruments, they allow for monitoring of changes during its clinical course and evaluating treatment outcomes in a precise and reproducible manner [4, 5].

Several facial function grading systems are available. Their usage, however, depends mainly on personal or institutional preferences. The House–Brackmann scale (H–B) was introduced in 1985 by the Facial Nerve Disorders Committee of the American Academy [6]. It is an ordinal scale which grades FP from 1 (normal function) to 6 (total paralysis). Although it has become the most commonly used grading system among otolaryngologists, it has been criticized for its low sensitivity to clinical changes in different regions of the face and for not evaluating synkinesis [5, 7–9].

Over the years, other scales addressing the limitations of the H–B scale have been developed and some of them have been adapted to new technology.

The Sunnybrook Facial Grading system (SFGS), described in 1996 by Ross et al. [10], is one of the most widely used scales in the world, especially among rehabilitation specialists. Facial symmetry at rest, voluntary facial movements, and synkinesis scores are measured separately and then used to produce a total score from 0 (total paralysis) to 100 (normal function). The Spanish version of the SFGS was recently validated by our group [11].

The electronic facial paralysis assessment (eFACE) was introduced in 2015 by Banks et al. from the Massachusetts Eye and Ear Infirmary [12]. It is an intuitive and reliable 16 item scale that uses visual analog scales to determine static and dynamic facial function, synkinesis, and total facial function symmetry, providing graphic and numerical results. It is administered using an application developed by Massachusetts Eye and Ear Infirmary and available on Apple devices (iPad or iPhone, iOS 6.0 or later). (Apple Inc. Cupertino, CA, USA) [7, 13].

Spanish is one of the most widely spoken languages in the world, with more than 500 million people using Spanish as their native language. In addition, more scientific papers are published in Spanish than any other language except English [14]. However, the eFACE has not yet been validated in Spanish, which hinders its adoption by Spanish-speaking professionals. The aim of the present study is to validate the Spanish version of the eFACE using cross-cultural adaptation and verifying its psychometric characteristics [15, 16].

## Methods

### Participants

This prospective study was conducted at the FP clinic at La Paz University Hospital in Madrid, Spain, between January and July of 2021. Inclusion criteria included Spanish-native adult patients with unilateral FP. All severity levels, time courses, and etiologies (central or peripheral) of FP were accepted. All participants provided written informed consent for study participation, including the collection of photographs and video recordings. Patients with bilateral palsy and those who did not consent to participate in the study were excluded. The study was approved by the Research Ethics Board of our Hospital (approval code PI-4599).

### Spanish eFACE

Prior to starting the study, express consent was obtained from the original author to translate the original eFACE to a Spanish language version.

**Fig. 1:**
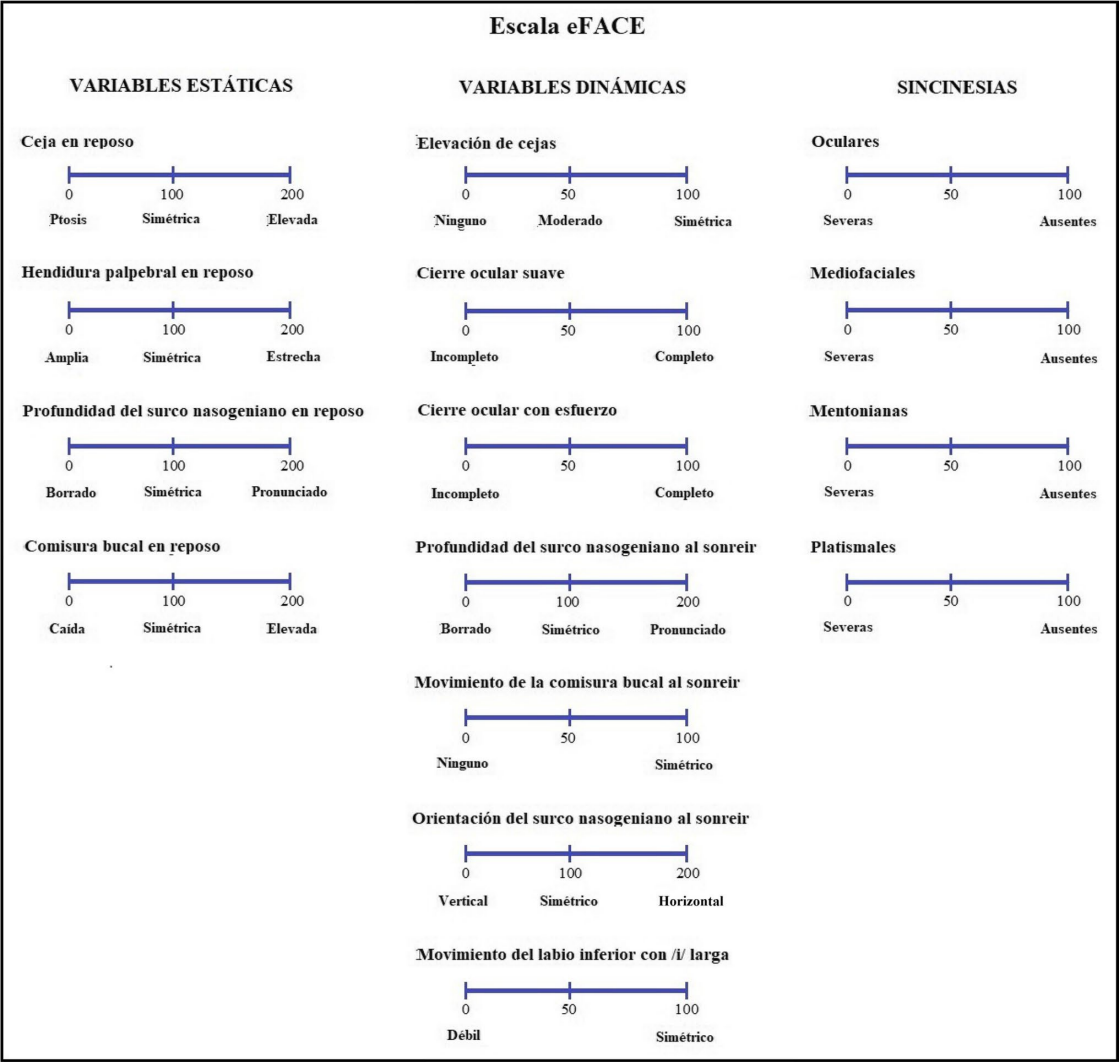
Spanish version of the eFACE scale

Cross-cultural adaptation of the Spanish language version was completed prior to study start [16, 17]. The original eFACE was translated into Spanish using a forward–backward translation method. The first translation was performed by two independent Spanish researchers who were fluent in both English and Spanish. The Spanish text was then back-translated into English by a native English-speaking translator blind to the original version. Significant discrepancies were resolved by reaching an agreement between researchers and translators, and a final Spanish version was established (Fig. 1).

Static facial symmetry was evaluated by observing the position of the eyebrow, the opening of the palpebral fissure, the depth of the nasolabial fold, and the position of the corner of the mouth with respect to the healthy side. Each item was scored from 0 to 200, with 100 corresponding to symmetry, 0 to complete asymmetry due to hypofunction, and 200 to complete asymmetry due to hyperfunction. Dynamic facial function was evaluated by observing the degree of muscle movement during eyebrow elevation, soft eye closure, straining eye closure, smiling, and pronunciation of the vowel sound /i/. Each item was scored from 0 to 100, with 0 corresponding to no movement and 100 corresponding to the symmetry between the healthy and the affected side. Smile analysis assessed the symmetry of the depth and orientation of the nasolabial fold, both of which were scored from 0 to 200, with 100 corresponding to symmetry, 0 to complete asymmetry due to hypofunction (blunted/vertical), and 200 to complete asymmetry due to hyperfunction (pronounced/horizontal). Finally, synkinesis was evaluated in four facial regions: ocular, midfacial, mentalis, and platysmal. Each region was scored from 0 to 100, with 0 corresponding to severe synkinesis and 100 corresponding to no synkinesis. Once each item was scored using a visual analog scale, the graphic and numerical results were automatically calculated by the application software. In this way, static, dynamic, synkinesis scores were obtained and a composite score was calculated, all of them in a range between 0 and 100.

Not all researchers used an Apple device (iPad or iPhone), so a Microsoft Access application (IBM Corp., New York, NY, USA) was developed by an engineer of the Department of Preventive Medicine to simulate the original eFACE application. The Microsoft Access application had the same visual analog scales, sliders for grading, automatic calculation of numerical results, and graphic output as the original application. It was available to be used on the computers with Microsoft software in the medical office.

### Video recording and evaluation of FP

The FP evaluation was made by five Spanish-native otolaryngologists with experience in FP assessment. The five raters had a training session prior to study start for approximately 2 h. For this, the original training video was watched, they evaluated individually the FP in 5 patients (who were not included in the study), and then, they met, shared, and discussed their results. That way, raters resolved any question about the procedure before collecting data from participants.

Videos and photographs were recorded during routine visits to the clinic in accordance with the standard recommendations of the Sir Charles Bell Society [18]. Photographs of ten standard static facial expressions and video recording of their respective dynamic functions were taken. All patients were recorded while sitting in the same position and in a room with the same lighting conditions and a uniform blue background. Based on the recorded images, the five raters, individually, graded the facial function of each patient using the H–B, the SFGS, and the Spanish eFACE in two independent sessions: the first one (t0) and the second one 2 weeks later (t1).

### Analyses

Descriptive variables are calculated. Continuous variables were presented as median and ranges. Discrete variables were presented as numbers and percentages.

Statistical analysis was performed in SPSS version 23.0 (IBM Corp., Armonk, NY, USA). Significance level was set at *p* < 0.05.

Reliability of the eFACE was determined using Cronbach’s α for internal consistency and intraclass correlation coefficient (ICC) for intra-rater and inter-rater agreement. An α coefficient of 0.70 or higher was considered reliable [19]. The ICC type A and type C were used to examine the intra- and inter-rater agreement [20]. An ICC 95% confidence interval (CI) was also used. In line with Landis and Koch [21], we considered the agreement to be “weak” if rated within 0–0.40, “moderate” within 0.41–0.60, “important” within 0.61–0.80, and “excellent” within 0.81–0.99.

The concurrent validity of the Spanish eFACE was established by comparing the scale with the H–B and SFGS, because these validated scales were used for psychometric analysis in the previous publications [22, 23]. Spearman’s rank correlation analysis was used to compare the H–B and the eFACE composite scores. Pearson’s correlation analysis was used to compare the SFGS and the eFACE subscales and composite scores. We considered correlations of 0–0.19 to be very weak, 0.20–0.39 to be weak, 0.40–0.59 to be moderate, 0.60–0.79 to be strong, and 0.80–1.00 to be very strong [24].

## Results

### Participants

Sixty-five patients met the inclusion criteria and were included in the study. There were 43 women and 22 men. Their age at presentation was 20–88 years (median: 51 years). 34 participants had FP on their right side (52.3%) and 31 had FP on their left side (47.7%). The time between the initial symptom and the first evaluation was 1–468 months (median: 15 months). The most common etiology of FP was Bell’s palsy (27.7%), followed by Ramsay–Hunt syndrome (18%), vestibular schwannoma surgery (16.9%), facial nerve tumor (6.2%) (2 hemangioma, 1 meningioma, and 1 not yet determined), post-traumatic palsy (4.6%), malignant neoplasia (3.1%) (1 squamous cell carcinoma, 1 salivary ductal carcinoma), and cholesteatoma (1.5%). 21.5% of participants had other FP etiologies. Regarding the management of FP, at the time of the evaluation, 44.6% had received treatment with corticosteroids (12.3% associated with antivirals); 33.9% underwent neuromuscular retraining therapy (26.2% associated with botulinum toxin) as their main treatment; and 21.5% had facial nerve reconstruction, either grafting (2 greater auricular nerve), or nerve transfer (4 hypoglossal-facial, 3 masseteric-facial, and 5 combined hypoglossal-facial and masseteric-facial transfers).

### The H–B and SFGS results

The H–B variable did not follow a normal distribution. Its scores ranged from grade 1 to 6 (both evaluations: median 3). At the first evaluation the H–B scores were: 2,5% grade I, 23,7% grade II, 31,4% grade III, 16,3% grade IV, 13,2% grade V, and 12,9% grade VI. At the second evaluation were: 1,2% grade I, 24,6% grade II, 33,2% grade III, 15,7% grade IV, 12,6% grade V, and 12,6% grade VI.

The SFGS variable follow a normal distribution. Its results ranged from 0 to 100 points (first evaluation: median 45, second evaluation: median 46).

### The eFACE scores

**Fig. 2:**
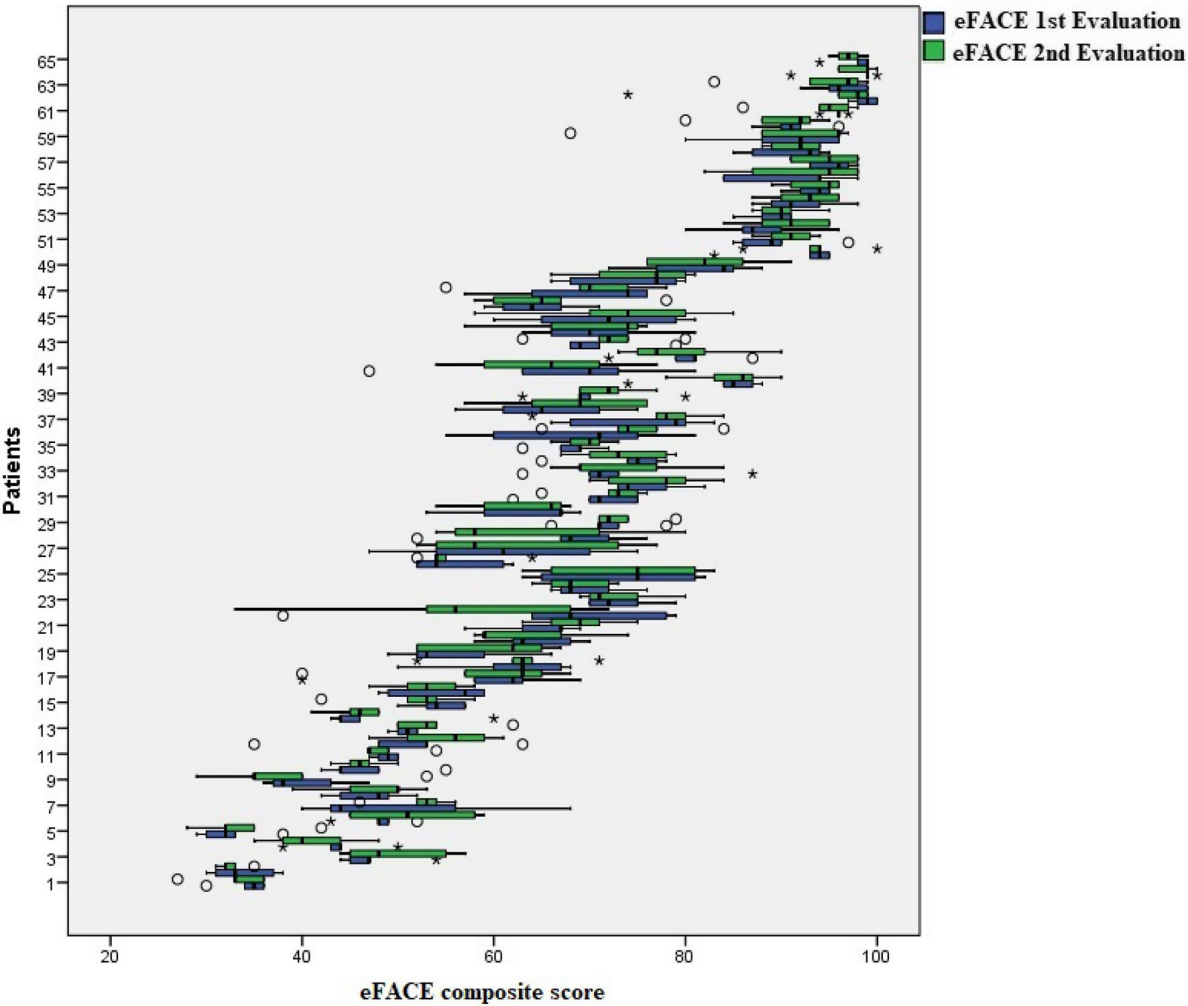
eFACE results. The composite scores for the 65 participants are presented as a boxplot. First (blue) and second (green) evaluations of each video by five evaluators are shown. Boxplots represent the minimum, first quartile, median, third quartile, and maximum of the eFACE composite scores. Circles are the outliers; asterisks are the extreme outliers. Higher scores correspond to better facial function

The eFACE variable followed a normal distribution. Its composite scores obtained from 65 participants by five raters in two evaluation sessions are shown in Fig. 2.

### Reliability of the Spanish eFACE

The internal consistency of the Spanish eFACE expressed as Cronbach’s α was 0.84 for the first evaluation session and 0.83 for the second session.

Based on the ICC results, the intra-rater agreement was excellent for the static, dynamic, synkinesis, and composite eFACE scores (Table 1). On synkinesis items, 2 of 5 raters had ICC scores of < 0.90 (0.81 and 0.79); otherwise, all raters had ICC scores of > 0.90 on the static, dynamic, and composite eFACE scores.

**Table 1:** Intra-rater agreement of the eFACE scale

	Composite		Static		Dynamic		Synkinesis	
	ICC	95% CI	ICC	95% CI	ICC	95% CI	IC**C**	95% CI
Rater 1	**0.96**	0.94–0.98	**0.95**	0.92–0.97	**0.97**	0.94–0.98	**0.95**	0.91–0.97
Rater 2	**0.99**	0.98–0.99	**0.96**	0.93–0.98	**0.99**	0.99–0.99	**0.92**	0.88–0.95
Rater 3	**0.96**	0.93–0.97	**0.92**	0.87–0.97	**0.97**	0.95–0.98	**0.81**	0.70–0.88
Rater 4	**0.95**	0.92–0.97	**0.95**	0.92–0.97	**0.96**	0.93–0.97	**0.79**	0.67–0.86
Rater 5	**0.96**	0.94–0.98	**0.94**	0.91–0.96	**0.96**	0.94–0.98	**0.96**	0.94–0.98

Bold values are the main values

*ICC* intraclass correlation coefficient type A (absolute agreement) for simple measures, *CI* confidence interval

At both evaluation sessions, the inter-rater agreement was excellent for the static, dynamic, and composite scores, and important for the synkinesis scores (Table 2). The mentalis synkinesis item had the lowest inter-rater correlation (0.46 for the first session and 0.45 for the second session). The eyebrow elevation item had the highest inter-rater correlation (0.96 for the first session and 0.95 for the second session).

**Table 2:** Inter-rater agreement of the eFACE scale

	1st evaluation		2nd evaluation	
	ICC	95% CI	ICC	95% CI
Static items	**0.86**	0.81–0.90	**0.86**	0.80–0.90
Brow at rest	0.76	0.68–0.83	0.75	0.67–0.83
Palpebral fissure at rest	0.74	0.65–0.81	0.68	0.59–0.77
Nasolabial fold depth at rest	0.85	0.79–0.90	0.83	0.77–0.89
Oral commissure at rest	0.82	0.76–0.88	0.81	0.75–0.87
Dynamic items	**0.93**	0.90–0.95	**0.93**	0.90–0.95
Eyebrow elevation	0.96	0.94–0.97	0.95	0.94–0.97
Gentle eye closure	0.87	0.82–0.91	0.86	0.81–0.91
Full eye closure	0.85	0.80–0.90	0.84	0.78–0.89
Nasolabial fold depth with smile	0.88	0.83–0.92	0.83	0.76–0.88
Oral commissure with smile	0.80	0.73–0.86	0.78	0.70–0.84
Nasolabial fold orientation with smile	0.63	0.53–0.73	0.64	0.53–0.73
Lower lip movement with /i/ sound	0.82	0.76–0.88	0.81	0.75–0.87
Synkinesis items	**0.69**	0.60–0.78	**0.65**	0.55–0.74
Ocular	0.63	0.53–0.73	0.62	0.51–0.72
Midfacial	0.75	0.67–0.82	0.76	0.68–0.83
Mentalis	0.46	0.35–0.58	0.45	0.34–0.57
Platysmal	0.64	0.54–0.74	0.62	0.52–0.72
Composite score	**0.90**	0.86–0.93	**0.89**	0.85–0.93

Bold values are the main values

*ICC* intraclass correlation coefficient type C (consistency) for simple measures, *CI* confidence interval

### Concurrent validity of the Spanish eFACE

The concurrent validity of the Spanish eFACE was established by comparing the scale with the H–B and SFGS scales. For all raters, very strong negative correlation was observed between the eFACE and the H–B (global results, *p* =  – 0.88 and  – 0.85 for each evaluation) (all *p* < 0.001).

A very strong positive correlation was found between the SFGS and the eFACE composite scores (*r* = 0.92 and 0.91 for each evaluation) (Table 3). A very strong positive correlation was also found for the dynamic scores (*r* = 0.94 and 0.93). A very strong negative correlation was found for the static scores at the first evaluation (*r* =  – 0.80) and synkinesis scores of the two scales (*r* =  – 0.88 and  – 0.89), with the exception of the static scores at the second evaluation, which had a strong negative correlation (*r* =  – 0.78) (all *p* < 0.001).

**Table 3:**
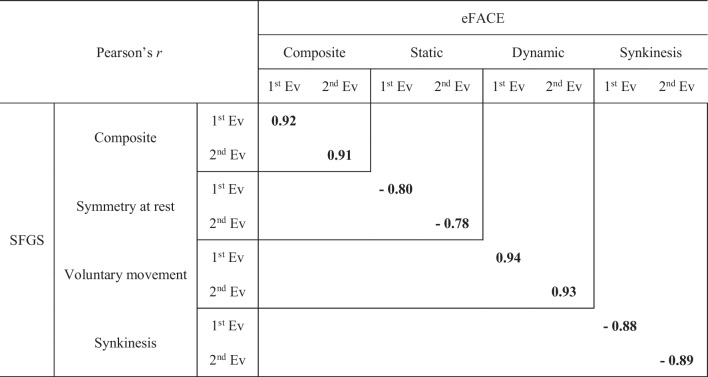
Concurrent validity of the eFACE scale vs the Sunnybrook Facial Grading System. Composite and subscale scores

For all results *p* < 0.001

*Ev* evaluation, *Pearson’s r* Pearson correlation coefficient, *SFGS* Sunnybrook Facial Grading System

## Discussion

### Main results

This study demonstrated that the Spanish language version of the eFACE is a reliable tool for evaluating patients with unilateral FP. The translated scale has a high internal consistency and excellent reproducibility based on the intra-rater agreement for the subscales and the composite score. The scale also has excellent concurrent validity, because it strongly correlated with other measures of FP, namely the H–B and SFGS.

### Evaluation of facial function in different languages

The absence of validated questionnaires on FP in many languages makes it difficult to obtain reliable and reproducible results. Even though Spanish is one of the most widely spoken languages in the world, facial function scales have not been validated in Spanish until recently. The Spanish version of the Facial Clinimetric Evaluation (FaCE) scale and the Facial Disability Index (FDI) scale were both validated in 2021, by Garcia-Iza et al. [25] and Gonzalez-Cardero et al. [26], respectively. The Spanish version of the SFGS was validated in June 2022 [11].

Since the emergence of the eFACE in 2015 [12], its use has increased, and according to Berner et al., it has become one of the most used clinical grading scales for evaluation of facial synkinesis, in addition to the H–B, the SFGS, and the Yanagihara scale [27]. Nevertheless, the majority of specialists that use this new tool are from English-speaking countries [3, 13, 22, 23, 27–31] and the number of publications by authors from other medical communities is lower, which is probably due to the idiomatic nature of English expressions used in the scale.

The original English eFACE has been implemented only on iOS devices, such as iPhone and iPad, which limits the potential target audience to Apple users. However, medical offices tend to have computers on which Microsoft Access is available, so the eFACE application was adapted to that platform. The availability of the eFACE across electronic devices helps to ease and speed up clinical evaluation. In addition, eFACE application calculates regional facial function assessment and static, dynamic, and synkinesis scores, which can then be exported to a database.

### Linguistic adaptation and validation of the Spanish version of eFACE

The adaptation and validation of the Spanish version of the eFACE included translating the scale into the Spanish language and verifying that this translation maintained the original’s psychometric properties. This study evaluated the psychometric properties of the Spanish version of the eFACE and provides support for its usage by professionals involved in the diagnosis and treatment of FP. Our results are in agreement with those presented by Banks et al. [13] and Chong et al. [22], who reported similar ICCs even among raters with different experience in FP evaluation.

### Reliability of the Spanish eFACE

In our study, the Spanish eFACE showed excellent reproducibility. The synkinesis score of one highly experienced rater was slightly less consistent [0.79, (0.67–0.86)], but it still showed an important intra-rater agreement. This may be attributed to the way in which the experienced evaluator based his evaluation more on his clinical experience and less on the specific instructions for the test [22].

The inter-rater agreement was excellent for the composite, static, and dynamic scores. Other authors have also reported excellent inter-rater agreement [12, 13, 22, 28]. The six key contributors to the global perceived disfigurement in FP suggested by Banks et al. are the nasolabial fold depth at rest, oral commissure position at rest, lower lip asymmetry while pronouncing /i/ sound, palpebral fissure width at rest, nasolabial fold orientation when smiling, and palpebral fissure width at full eye closure [28]. All of these have important-to-excellent reliability in our study. These items belong to the static and dynamic subscales, with voluntary eyebrow elevation having the highest inter-rater reliability both in this study and in Banks et al. Our study also reproduced the weaker agreement for the synkinesis score reported by Banks et al. However, we found that mentalis synkinesis showed the lowest inter-rater agreement [ICC 0.46, (0.34–0.58)] in contrast to platysmal synkinesis in Banks et al. This difference could be because both movements occur in the lower third of the face and their evaluation could be affected by the accuracy, the depth of field, or shadowing of the images [7]. Synkinesis items are usually the most difficult to assess irrespective of the scale used [11, 32]. When evaluating synkinesis, not all raters may focus on the same region because they have to look at the whole face (unlike voluntary movement evaluation, when the rater’s attention is directed to a specific muscle).

### Correlation between the eFACE and the H–B

A very strong negative correlation (*p* < 0.001) was established when comparing the eFACE composite score with the H–B grade. The negative association was expected, because higher FP severity corresponds to higher H–B grades but lower eFACE scores. The H–B is considered the standard FP grading system, and, as such, it was used as a reference in other FP scale validation studies [22]. Therefore, our finding supports the validity of the Spanish eFACE.

### Correlation between the eFACE and the SFGS

A very strong correlation (*p* < 0.001) was obtained between the eFACE and the SFGS in the subscales and in the composite scores. The correlations were higher than those obtained in the previous studies [22, 23]. The negative association in the static and synkinesis subscales was expected, because for these subscales, higher FP severity corresponds to higher SFGS subscale scores but lower eFACE subscale scores.

The only strong negative correlation (*r* =  – 0.78) was obtained in the static subscale. This lower correlation between the static eFACE and SFGS subscales was also observed by Chong et al. [22] and Gaudin et al. [23]. Even though both scales measure similar aspects of static facial function, they do so differently. For example, when evaluating the palpebral fissure, history of eye surgery worsens the static SFGS score regardless of the surgery outcome, whereas the eFACE measures the actual asymmetry with respect to the healthy side [30]. This makes the eFACE a better scale to evaluate surgical results in the ocular region.

### Advantages and limitations of the eFACE

Sliding visual analogue scales and automatic calculations on digital devices reduce human error and are easy-to-use. Another advantage of the eFACE is immediate visualization of results, which facilitates the evaluation of FP changes over time.

The eFACE and the SFGS assess static, dynamic, and synkinesis parameters of facial function using continuous numerical scales, while the H–B uses only a single global ordinal scale. In addition, the eFACE evaluates both flaccidity and hypertonia of different facial regions. For these reasons, the eFACE appears to be highly useful for evaluating the results of facial function reconstruction [29, 30].

However, the eFACE, as well as the SFGS and the HB, remains a subjective instrument. Natural beauty, make-up, head position, and lighting may influence clinician’s perception of the facial dysfunction. To minimize subjectivity, facial evaluation should be carried out always in optimal conditions. Attempts to introduce objective tools have had limited results. On the one hand, congenital malformations and acquired asymmetries, such as grafts or scars, may preclude normal measurements of a static face. On the other hand, evaluating facial motion is more complicated and so far, no objective tool (e.g., optical scanners or mapping cameras) has achieved universal acceptance for everyday clinical practice. There are some promising tools as Emotrics or Auto-eFACE, based on automatic assessment of facial function, which may eliminate observer bias. Until a universally accepted objective tool is developed, we still need to rely on subjective scales that allow us to monitor changes in facial function, and therefore, it is important to validate these scales to obtain reliable results [33–36].

The perfect tool to assess FP would be easy and quick to use on any portable electronic device and automatically provide numerical and graphic results, and record and objectively evaluate static, dynamic, and synkinesis parameters in both flaccid and non-flaccid FP. Until such instrument is developed, the eFACE remains an intuitive and easy-to-use scale that has demonstrated good reliability and reproducibility in English and Spanish.

## Conclusion

The study demonstrated that the Spanish language version of the eFACE has high reliability and validity. This tool appears to be useful for Spanish-speaking physicians involved in the diagnosis, treatment, and follow-up of patients with FP. The use of reliable and validated assessment tools in Spanish is fundamental for improving communication between professionals and producing high-quality studies.

## Data Availability

The data presented in this study are available on reasonable request from the corresponding author.
